# Effects of rainfall manipulation and nitrogen addition on plant biomass allocation in a semiarid sandy grassland

**DOI:** 10.1038/s41598-020-65922-0

**Published:** 2020-06-03

**Authors:** Jing Zhang, Xiaoan Zuo, Xueyong Zhao, Jianxia Ma, Eduardo Medina-Roldán

**Affiliations:** 1 Northwest Institute of Eco-Environment and Resources, Chinese Academy of Sciences, Lanzhou, 730000 China; 2 Health and Environmental Science Department, Xi’an Jiaotong Liverpool University, Suzhou, 215123 China

**Keywords:** Boreal ecology, Plant ecology, Grassland ecology

## Abstract

Extreme climate events and nitrogen (N) deposition are increasingly affecting the structure and function of terrestrial ecosystems. However, the response of plant biomass to variations to these global change drivers is still unclear in semi-arid regions, especially in degraded sandy grasslands. In this study, a manipulative field experiment run over two years (from 2017 to 2018) was conducted to examine the effect of rainfall alteration and nitrogen addition on biomass allocation of annuals and perennial plants in Horqin sandy grassland, Northern China. Our experiment simulated extreme rainfall and extreme drought (a 60% reduction or increment in the growing season rainfall with respect to a control background) and N addition (20 g/m^2^) during the growing seasons. We found that the sufficient rainfall during late July and August compensates for biomass losses caused by insufficient water in May and June. When rainfall distribution is relatively uniform during the growing season, extreme rainfall increased aboveground biomass (AGB) and belowground biomass (BGB) of annuals, while extreme drought reduced AGB and BGB of perennials. Rainfall alteration had no significant impacts on the root-shoot ratio (R/S) of sandy grassland plants, while N addition reduced R/S of grassland species when there was sufficient rainfall in the early growing season. The biomass of annuals was more sensitive to rainfall alteration and nitrogen addition than the biomass of perennials. Our findings emphasize the importance of monthly rainfall distribution patterns during the growing season, which not only directly affect the growth and development of grassland plants, but also affect the nitrogen availability of grassland plants.

## Introduction

Climate change and excessive human activity changed the rainfall patterns and increased the emissions of bioactive nitrogen (N) into the atmosphere, which have produced profound impacts on the global rainfall and N cycle^1,2^. Rainfall and N are key environmental factor determining ecosystem structure and function, especially in water and N limited grasslands^3^. Therefore, changes in rainfall and N may have a strong impact on terrestrial ecosystem and may feed back into climate change.

Plant biomass allocation refers to the distribution of limited resources by plants in order to maximize the benefits of growth, maintenance and reproduction in response to environmental clues having profound implications for plant growth and development^4^. Increasing rainfall can generally promote the accumulation of aboveground biomass while decreasing rainfall can promote the growth of root^5^. However, the accumulation of aboveground and belowground biomass is not synchronous^6^. Plants also adapt to drought by increasing the root-shoot ratio (R/S)^7^. However, some studies have also found a negative correlation between rainfall and biomass, as rainfall increases soil erosion and decreases soil organic matter content, thus reducing grassland productivity^8^. In summary, the effect of rainfall variation on biomass allocation varies with rainfall gradients, elevation gradient and species composition^9^.

Changes in global water circulation are forecast to enhance both inter- and intra-annual variability of rainfall^10,11^. Chinese scientists forecast that rainfall patterns will be more complicated and multifrequency in the next 30 years in north of China^12^, and this will produce more frequent rainfall and drought events in the future^13^. Studies of plant biomass have focused mainly on the effect of total annual rainfall^14^, but recent research showed that rainfall at a particular time of the year (seasonal rainfall) can explain biomass changes better than total rainfall^16,17^ due to the different water requirements of terrestrial plants at different growth stages^15,18^. Studies have showed that advanced rainfall during the growing period can lead to an improvement in the utilization of soil water, which is beneficial to root growth^19^. By contrast, delayed rainfall often increases the availability of soil water at the later stage of growth, thus delaying the plant senescence^20^. However, it is less known how excessive rainfall and extreme drought affect biomass allocation among years with different rainfall distribution during the growing season.

Nitrogen inputs to the earth’s ecosystems are increasing, and this will have profound impacts on the function of terrestrial ecosystems^21^. For instance, during the period 1990–2003, atmospheric N deposition increased dramatically from 8.7 kg N ha ^−1^ to 13.8 kg N ha ^−1^ ^22^. Many studies showed that increased amounts of available N decreased root biomass and R/S^23^. The effect of N on grassland species is determined by rainfall conditions, and increasing soil N availability increased water absorption capacity of plants^23–25^. However, little is known with respect to how rainfall changes, N deposition and their interaction in semiarid grasslands will affect the allocation of aboveground and belowground biomass distribution of annuals and perennials.

In semi-arid regions, the vegetation biomass is most sensitive to water availability because rainfall is concentrated in the plant’s growing period^26^. Therefore, rainfall events with long duration and copious amounts during the growing season may play a primary role in regulating the growth of vegetation^13,27^. Semiarid ecosystems such as the Horqin sandy grasslands in Inner Mongolia may be particularly susceptible to N deposition and climate change as the region is largely constrained by both resources^28^. Annuals and perennials are the main herbaceous plants in the Horqin sandy grassland. Annuals have a fast-growing strategy (e.g., shallower/fine roots), which allows plants to rapidly absorb water and nutrients under favorable conditions to complete their life cycle^29^. Compared to annuals, perennials have a stronger ability of survival and competition in nutrient-poor and water-deficient environments because of their deeper intricate root system^30^. So annuals are more likely to survive in harsh environment than perennials, and exploring the biomass allocation strategies of annuals and perennials can be a powerful predictor for environmental change. In the context of global climate change, conducting research through coupled rainfall-nitrogen experiments is of great significance for a deeper understanding of adaptation mechanisms of degraded grassland species to climate change drivers. In this study, we investigated aboveground biomass (AGB), belowground biomass (BGB), coarse roots biomass (CR) and fine roots biomass (FR) of annuals and perennials in a grassland community under different rainfall manipulation and nitrogen treatments. We addressed the following questions: (1) how do AGB, BGB, CR and FR of annuals and perennials change with rainfall alterations and nitrogen addition? (2) How do the allocation of AGB and BGB between annuals and perennials differ in their response to changes in rainfall and N?

## Materials and Methods

### Study area

This study was carried out in Horqin sandy grassland near the Naiman Desertification Research Station, Chinese Academy of Science. The climate is typical semiarid continental seasonal monsoon. The average annual rainfall is 360 mm, and nearly 75% is concentrated in the growing season (May to August)^31^. The soil is aeolian sandy soil according to the Chinese soil taxonomy classification system (http://www.resdc.cn).

### Experimental design and measuring

The experiment was conducted between 2017 and 2018 on sandy grassland which was relatively homogeneous and not so severely degraded. Based on the long-term observation data of total annual rainfall, extreme rainfall and extreme drought events during the growing season in this region^32^, we set up the extreme rainfall and extreme drought treatments as follows: a 60% increment (with respect to background rainfall) of rainfall during the growing season from May 1 to August 31 (extreme rainfall), and a 60% reduction of rainfall during the growing season from May 1 to August 31 (extreme drought).

**Figure 1:**
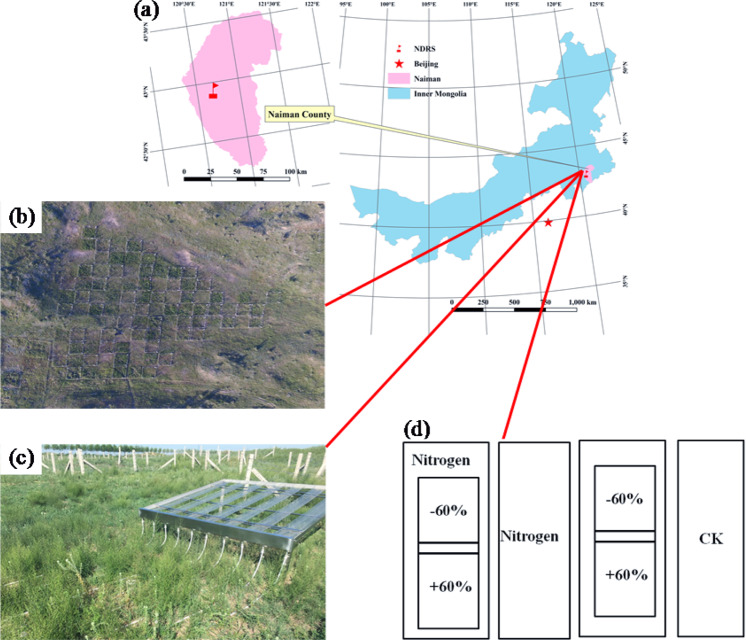
Location of the study area (**a**), aerial photograph of the field site (**b**), rainfall shelters installation (**c**) and experiment design (**d**). An artificial simulation of rainfall device in the field experiments to achieve the goal of increasing or decreasing rainfall: (**c**) ±60%: increasing or decreasing rainfall by 60% during the growing season (May to August).

In order to simulate the effect of N deposition, we adopted a rather high N addition level (20 g nitrogen / m^2^^2^) in our experiment. There are three reasons we used this level of N addition: First, the sandy grassland is relatively barren^33^, thus in order to achieve a better experimental effect, the amount of added N needs to be high. Second, the Horqin sandy land is located in the ecologically fragile zone of semi-arid farming-pastoral interlacing area in Northern China. Large-scale human activities (farmland fertilization and animal husbandry) in this area have intensified the N input, resulting in excessive N load in the area^34^. Besides, by referring to N deposition levels in some countries around the world (e.g., USA and Europe)^35^, a 20 g N / m^2^^2^ rate is deemed representative of the highest deposition levels in larger areas of China^36^. Thus in this experiment, 10 g N / m^2^ were added sequentially both in May and July in 2017 and 2018. Six treatments (with 6 replicates each) were randomly arranged for the interaction of rainfall change and N addition. The treatment included control (CK), + 60%, −60%, +N, + 60% × +N, −60% × +N (Fig. 1d). The rainfall alteration device (rainfall shelters) was firstly described by Yahdjian and Sala^28^, and has been widely used in research of climate change, owing to its low cost and minimal influence on the microclimate^37,38^. Each rainfall shelter was made of clear polycarbonate plastic strips that allow 90% sunshine penetration to ensure no great alterations on plant photosynthesis (Fig. 1c).

In mid-August of 2017 and 2018, we selected a representative land with evenly distributed vegetation; containing as many species as possible. For each plot one soil cube of 30 × 30 × 30 cm was excavated with whole species in each of the plots. The samples were gently shaken to get rid of the soil particles attached to roots, and then brought to the laboratory for washing off the remained soil particles. The AGB and BGB of each of plant species were measured. Moreover, BGB was separated into CR (diameter greater than 2 mm) and FR (diameter less than 2 mm) by the vernier caliper^39^. The separated biomass was oven-dried at 85 °C for 48 h to obtain dry weight.

### Data analysis

The slope of the rainfall-biomass relationship reflects the sensitivity of biomass to rainfall variability. A sensitivity of 1 indicates that a relative change in response to parameters in the same direction^28^. Sensitivity was calculated by the relative change in the rainfall manipulation or N addition plots in comparison to the control plots as:

1 $${\rm{S}}{\rm{e}}{\rm{n}}{\rm{s}}{\rm{i}}{\rm{t}}{\rm{i}}{\rm{v}}{\rm{i}}{\rm{t}}{\rm{y}}=\frac{|\mathrm{biomas}{s}_{\mathrm{control}}-\mathrm{biomas}{s}_{\overline{\mathrm{ck}}}|}{\mathrm{biomas}{s}_{\overline{\mathrm{ck}}}}$$

Where $biomas{s}_{control}$ and $biomas{s}_{\overline{ck}}$ represents the biomass of plants under either a control plot or a treatment plot respectively. The higher the value of sensitivity the higher the sensitivity of biomass to changes in variation in rainfall.

Three-way ANOVA was used to test the effects of rainfall pattern, nitrogen addition impact on the AGB, BGB, CR and FR in the two different years. Results were considered significantly different at a *P* < 0.05 level. Data analysis and plotting were carried out by SPSS 21.0 and SigmaPlot 12.5, respectively.

## Results

Rainfall manipulation and nitrogen addition in different years had significant effects on AGB, BGB, CR and FR of annuals and perennials in sandy grassland, but the interactive effects of rainfall and nitrogen had no significant effect on biomass (Table 1).

**Table 1:** F values of three-way ANOVAs of community with rainfall (R), nitrogen addition (N) and year (Y).

	AGB		BGB		CR		FR	
	F	Sig.	F	Sig.	F	Sig.	F	Sig.
	community							
R	12.281	0.000	4.945	0.001	0.834	0.506	5.100	0.001
N	0.014	0.907	8.048	0.006	2.343	0.129	5.700	0.019
Y	9.684	0.002	7.053	0.009	0.821	0.367	7.495	0.007
R × N	0.258	0.904	0.639	0.636	0.216	0.929	0.807	0.524
R × Y	2.820	0.029	0.538	0.708	1.243	0.298	0.845	0.5000
N × Y	8.436	0.005	0.617	0.434	1.024	0.314	0.01	0.921
R × N × Y	1.319	0.268	0.297	0.879	0.645	0.632	0.145	0.965
	annuals							
R	4.947	0.002	4.274	0.004	0.86	0.493	4.328	0.004
N	0.864	0.356	0.342	0.561	10.757	0.002	0.055	0.816
Y	1.316	0.256	0.41	0.524	6.998	0.01	1.988	0.164
R × N	0.055	0.994	0.105	0.98	0.643	0.634	0.173	0.951
R × Y	2.592	0.046	3.736	0.009	0.409	0.801	4.661	0.002
N × Y	7.918	0.007	1.28	0.262	8.738	0.004	0.205	0.652
R × N × Y	0.934	0.451	0.431	0.786	1.298	0.281	0.383	0.820
	perennials							
R	2.965	0.024	3.014	0.022	0.997	0.414	2.622	0.040
N	7.834	0.006	2.149	0.146	0.015	0.904	4.308	0.041
Y	0.587	0.446	0.034	0.855	1.648	0.203	5.14	0.026
R × N	0.539	0.708	0.885	0.477	0.558	0.693	0.741	0.566
R × Y	0.548	0.701	1.284	0.283	0.585	0.674	1.609	0.180
N × Y	1.906	0.171	0.664	0.418	2.292	0.134	0.103	0.750
R × N × Y	0.357	0.839	0.112	0.978	0.976	0.425	0.118	0.976

AGB: Aboveground biomass; BGB: belowground biomass; CR: coarse roots; FR: fine roots. *P < 0.05, **P < 0.01.

### Rainfall change

Overall, the total rainfall during the growing season did not differed much between 2017 and 2018, but the monthly distribution of rainfall was quite different between the two years. In this way the rainfall amount in the early period of the growing season (May to June) in 2017 was 41.8 mm; only half of that in the early time of the growing season in 2018 (100.8 mm). The rainfall in the late period of the growing season (July to August) in 2017 was 232.6 mm, while in the late period of the growing season in 2018 was just 136.6 mm (Table 2).

**Table 2:** Total rainfall and monthly rainfall during the growing season.

Rainfall(mm)	2017			2018		
	CK	−60%	60%	CK	−60%	60%
Total	274.4	109.8	439.04	236.37	94.55	378.19
May	34.4	13.76	55.04	17.65	7.06	28.24
Jun	7.4	2.96	11.84	83.17	33.27	133.07
July	91	36.4	145.6	47.65	19.06	76.24
August	141.6	56.64	226.56	87.9	35.16	140.64

CK: background rainfall; ±60%: increasing or decreasing rainfall by 60% during the growing season (May to August).

**Figure 2:**
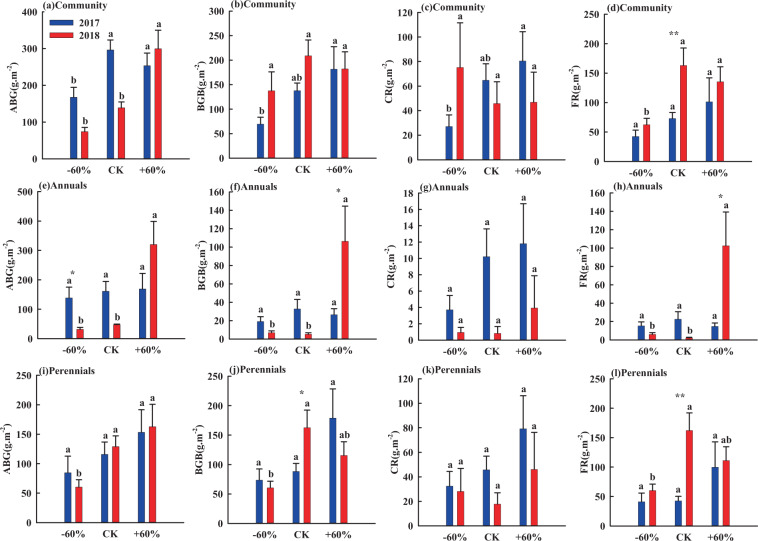
Influence of rainfall alteration on the biomass of grassland plants. Different values represent mean ± SE. Different letters indicate significant differences between rainfall treatments in the same year (*P* < 0.05). Significant differences between 2017 and 2018 are indicated by asterisks, *P < 0.05, **P < 0.01.

At the grassland community level, extreme drought (-60%) significantly reduced AGB by 44% in 2017, while extreme rainfall (+60%) significantly enhanced it by 115% in 2018 (Fig. 2a). On the other hand, extreme rainfall and extreme drought had no significant effects on the BGB and CR in 2017 and 2018 (Fig. 2b,c). Extreme rainfall and extreme drought had no significant effects on FR in 2017, while extreme drought significantly reduced FR in 2018 by 62% (Fig. 2d). Extreme rainfall and extreme drought had no significant effects on AGB, BGB, CR and FR in 2017 for neither annuals nor perennials, (Fig. 2e–l). On the other hand, extreme rainfall significantly enhanced AGB, BGB and FR of annuals by 579%, 202% and 800% respectively in 2018 (Fig. 2e,f,h), and extreme drought significantly reduced the AGB and BGB and FR of perennials by 53%, 63% and 63% respectively in 2018 (Fig. 2i,j,l). Extreme rainfall and extreme drought had no significant effects on CR of annuals and perennials in both of 2017 and 2018 (Fig. 2g,k).

### Nitrogen addition

**Figure 3:**
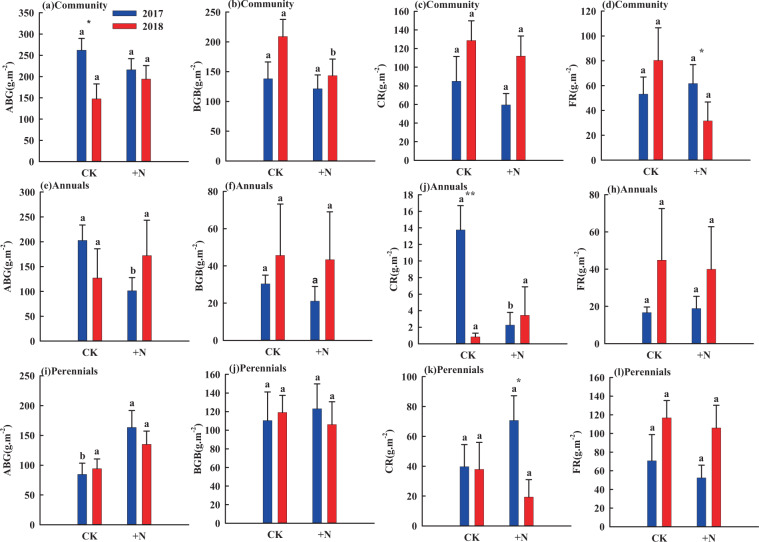
The influence of nitrogen addition on biomass of grassland plants. Different letters indicate significant differences between nitrogen treatments in the same year (*P* < 0.05). Significant differences between 2017 and 2018 are indicated by asterisks, *P < 0.05, **P < 0.01.

For the whole grassland community, N addition had no significant effects on AGB, BGB, CR and FR in 2017 (Fig. 3a–d), but it decreased BGB by 32% in 2018 (Fig. 3b). In 2017, N addition significantly decreased AGB and CR of annuals by 50% and 85% respectively (Fig. 3e,j), while it enhanced AGB by 87% of perennials (Fig. 3i). In 2018, N addition had no significant effect on AGB, BGB, CR and FR of neither annuals nor perennials (Fig. 3e–l).

### R/S

**Figure 4:**
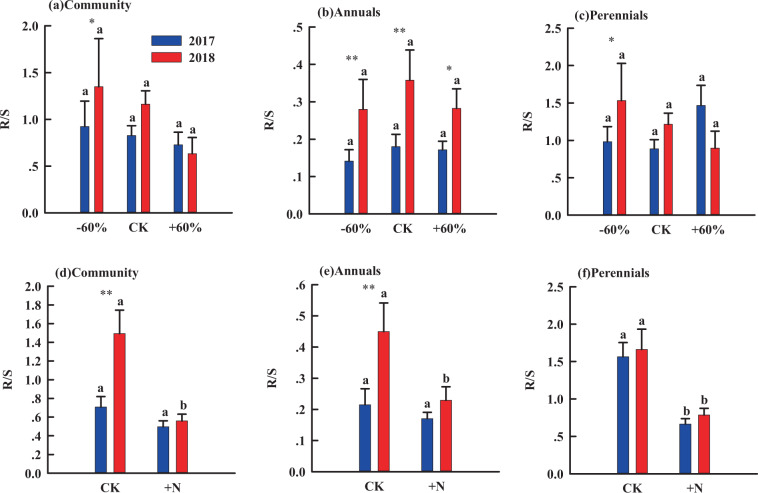
The effect of rainfall change and nitrogen addition on the root-shoot ratio (R/S). Different letters indicate significant differences between nitrogen treatments in the same year (*P* < 0.05). Significant differences between 2017 and 2018 are indicated by asterisks, *P < 0.05, **P < 0.01.

The R/S of community, annuals and perennials had different responses to rainfall alteration and N addition (Fig. 4). Rainfall change had no significant effects on the R/S of the community, annuals or perennials (Fig. 4a–c). Under extreme drought, R/S of the plant community, annuals and perennials varied significantly between the two years. Besides, the R/S of annuals was significantly different in two years with different rainfall patterns. In this way, N addition significantly decreased the R/S by 56% of perennials in 2017(Fig. 4f), and it significantly decreased the R/S of community, annuals and perennials by 64%, 50% and 51% respectively in 2018(Fig. 4d–f).

### Sensitivity

**Figure 5:**
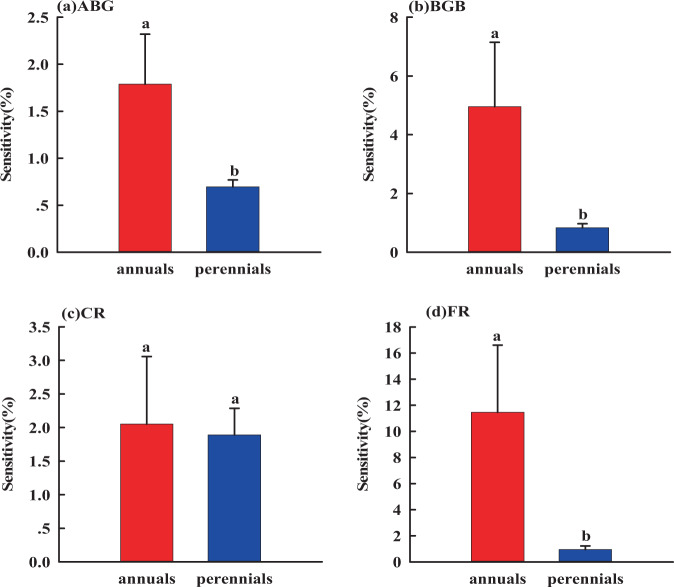
Sensitivities of AGB, BGB, CR and FR of grassland plants to rainfall change. Different letters represent the difference in nitrogen sensitivity between annuals and perennials (*P* < 0.05).

**Figure 6:**
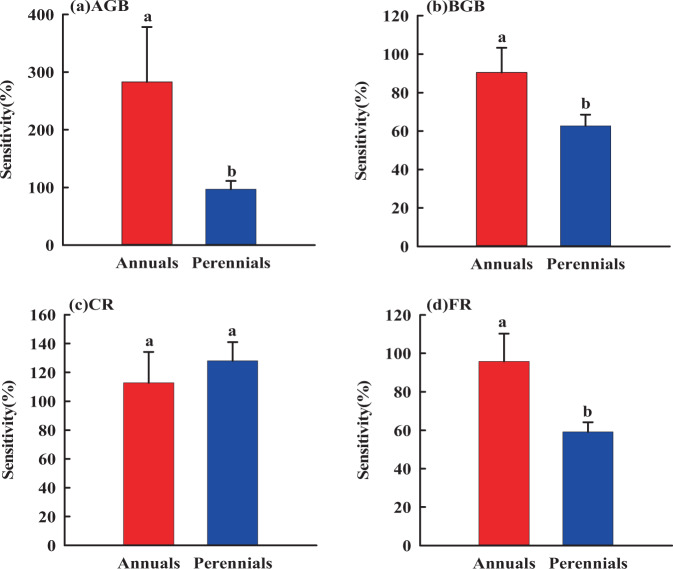
Sensitivities of AGB, BGB, CR and FR of grassland plants to nitrogen addition. Different letters represent the difference in nitrogen sensitivity between annuals and perennials (*P* < 0.05).

Sensitivities of AGB, BGB and FR in response to rainfall alteration varied in the annuals and perennials (Fig. 5,6). Specifically, AGB, BGB and FR of the annuals were more sensitive than those of perennials to rainfall changes (Fig. 5a,b,d). Similarly, AGB, BGB and FR of the annuals were more sensitive than those of perennials to N addition (Fig. 6a,b,d). However, CR of the annuals and perennials was not sensitive to rainfall change and N addition (Fig. 5c,6c). In summary, the biomass of annuals was more sensitive than the biomass of perennials to rainfall change and N addition.

## Discussion

### Rainfall

In the present study, extreme drought decreased AGB of the whole grassland community in 2017, while extreme rainfall enhanced AGB of grassland community in 2018 (Fig. 2a–d). These results demonstrate a clear pattern that increased rainfall significantly increases the biomass of grassland species, while decreased rainfall significantly decreases the biomass of grassland species. The result is consistent with long-term observations of terrestrial ecosystems around the world^40^. In addition, our results show that although total rainfall during the growing season of 2017 and 2018 was almost equal, the effects of extreme rainfall and extreme drought on AGB, BGB, CR and FR were greatly different between the two years (Table 1; Fig. 2). This result further confirms previous research which has showed that the seasonal distribution and intensity of rainfall, rather than total rainfall determine grassland productivity^16,18^.

Plants in arid environments show a delayed phenology to reduce water loss^41^. This response mechanism occurs under drought conditions, indicating that plants have begun to enter into dormancy, which continues into the next rainfall season, thus forming a strategy to improve survival. In this way, longer intervals between rainfall events shorten the growing season fort plants^42^. In addition, the recovery of plants after the dormant period is closely related to the interval and intensity of rainfall after drought^43^. Our results further support this view: rainfall change had impacts on the biomass of both annuals and perennials in 2018, but not in 2017 (Fig. 2e–f). This differential response of biomass was mainly caused by rainfall differences in the early time of growing season (May to June) between the two years. Thus, in 2017, prolonged drought in May and June delayed the germination of grass seeds. Until July, extreme drought did not affect the normal growth and development of grassland plants, because sufficient rainfall in July and August exceeded the rain threshold needed for plant growth, so the 60% decreased rainfall treatment could also meet the requirements for normal growth of sandy grassland plants^14,18^. These results showed that in late July and August, rainfall compensates for biomass losses caused by insufficient water in early stages of the growing season. Thus, a period of sufficient rainfall during the growing season may play a more important role in promoting biomass accumulation. These findings are inconsistent with studies in other places where rainfall in later periods in the growing season has been associated with a higher risk of microclimate moisture, bacterial invasion, and a higher risk of soil compaction^44^. The main reason for discrepancies in our results and those of previous studies is the difference of environmental factors (temperature, moisture, elevation, etc.) in different regions. Sandy soil has a large number of large particles, higher soil saturated hydraulic conductivity and evaporation, so the soil water content decreases rapidly after rainfall events due to its low water-holding capacity^45^. Therefore, rainfall later in the growing season will not harm the ecosystem structure of sandy grassland.

The rainfall was distributed more evenly during the growing season in 2018. Therefore, extreme rainfall or extreme drought would be expected to have positive or negative impacts on vegetation biomass accordingly. Extreme rainfall significantly increased the AGB and BGB of the annuals, and extreme drought significantly reduced the AGB and BGB of perennials (Fig. 2e–l). This was mainly due to the fact that the shallower roots of annuals can use water quickly and complete their life cycle rapidly under favorable water conditions^45^. Therefore, increasing rainfall by 60% throughout the growing season can effectively increase the AGB and BGB of annuals. Although perennials have a well developed and deep root system, because of the coarse texture of the soil and its lower water-holding capacity, extreme drought dramatically reduced the effective moisture of sandy soil^45^. This, the roots of perennials could not obtain enough water from deep soil layers, and the AGB and BGB of perennials were synchronously decreased^14^.

### Nitrogen

As a limiting nutrient in semi-arid regions, N has a great influence on plant growth^46^. Our results showed that in 2017, N addition decreased the AGB of annuals, while increased the AGB of perennials (Fig. 3e, i). This is mainly because the water deficit in the early growing season in 2017 made N the main limiting element for plant growth. While added N eliminated nutrient limitation and turned plants’ competition for nutrients into competition for other resource such as light or water^32,47^. Taller perennials had a competitive advantage over light resource. In addition, the developed root system of perennials also provided them with a competitive advantage for water resources. As perennial individuals grew larger, they would devote more biomass to photosynthetic organs (e.g. stem and leaf) to enhance productivity^48^. On the contrary, annuals lose their competitiveness for light and water resources and their AGB decreased accordingly^49^. This is consistent with previous studies where a reduction in biomass of some plant species was compensated by an increase in biomass of other plant species in the plant community^29,30^.

### R/S

Isometric allocation hypothesis demonstrated that AGB scales one-to-one with respect to BGB among different kinds of plants and this relationship is insensitive to changes in environmental conditions^50,51^. Other researchers have proposed optimal partitioning theory, which suggests that plants preferentially allocate biomass to the organ that is more efficient in obtaining resources^4,50^. In this study, rainfall changes had no significant effects on the R/S of community, annuals and perennials in 2017 and 2018 (Fig. 4a–c). These results are inconsistent with previous research showing that plants often allocated more biomass resources to aboveground reproductive organs to further improve reproduction and photosynthetic capacity when rainfall is abundant^51^, or plants often allocate more biomass to belowground in response to extreme drought^52^. One possible explanation may be that the allocation of plant biomass depends largely on the size of the plant itself, rather than the external environment^53^. Another explanation is that R/S of sandy soil species may take longer time to respond to rainfall change. As previous studies have shown rainfall changes do affect the allocation of plant biomass, but these responses become apparent only 10 years after the experimental manipulation^54^. Although rainfall change had no significant effect on R/S, R/S did vary significantly between the two years, especially in the extreme drought condition. This suggests that under extreme drought conditions, AGB and BGB decreased synchronously. These conclusions verify the role of compensatory interactions among sandy grassland plants, and suggest that competition of plants in the sandy grassland community would result in a trade-off between annuals and perennials^55^.

In addition, we found that N addition had no effect on R/S of the whole grassland community in 2017, while it decreased R/S of the whole grassland community in 2018 (Fig. 4d–f). The main reason for this result is the difference in rainfall distribution. In 2018, N stimulated by abundant water caused soil acidification^56^, which causes the roots of plants to be exposed to a high concentration and toxicity of protons (H^+^), aluminum (Al_3_^+^) as well as alteration of resource stoichiometry^57^. These effects directly lead to a decrease in the respiration rate of soil microorganisms, partially offsetting root respiration and thus reducing BGB^56^. This is consistent with the results of previous studies, where the responses of different microbial to N addition are likely due to different soil water content^58^. These results were partly consistent with other grassland manipulative experiments, which suggests that in Horqin sandy grasslands, the coupling effects of rainfall and N early in the growing season may have a negative effect on the growth of plant roots. This further explains why nitrogen-water coupling has no significant effect on the growth of sandy grassland community, annuals and perennials (Table 1), indicating that decoupling of root morphology and their water uptake with increasing soil N availability.

### Sensitivity

Our study showed that the R/S of annuals was significantly different in two years with different rainfall patterns (Fig. 4b), and AGB, BGB and FR of annuals were more sensitive to rainfall change and nitrogen addition than that of perennials (Fig. 5–6), which was consistent with the findings that annuals can adjust nutrient allocation much more faster than the perennials to complete their life cycle across favorable water and fertilizer conditions^47,51,53^. Similarly, we also have found that fine root was more sensitive to rainfall change and nitrogen addition than coarse roots. This can be explained by that coarse roots have little effect on absorption function^59^, while fine root is closely related to soil water and nutrients and is the main organ of water and nutrient absorption^60^. From the above, we are convinced that the sensitivity of annuals is mainly caused by their large number of fine roots.

In two-year study, we found that extreme rainfall and extreme drought have produced multiple effects on sandy grassland plants. It will be necessary to repeat our study in a year with more uniform rainfall and with a longer duration to see whether and how the results change. This study will help us to predict the impacts of climate change and make feasible decision for sustainable restoration and management of the degraded sandy grassland.

## Conclusions

This study explored the biomass allocation of sandy grassland in semiarid regions and its relationships with environmental factors. We found that the sufficient rainfall in late growth season would promote or compensates for biomass losses caused by insufficient water in early growth season. In the years with more uniform rainfall during the growing season, extreme rainfall increased the biomass of annuals, and extreme drought decreased the biomass of perennials. R/S of sandy grassland plants was not affected by rainfall change, but coupling effects of rainfall and nitrogen in early growing season can reduce R/S of grassland plants. Along with total rainfall during the growing season is increasingly used to explain ecosystem processes, we argue that the distribution pattern of rainfall will have more profound impacts on the distribution of plant biomass in semi-arid grassland. The result of this research is of great importance for deserts plants adapt to global changes.

## Supplementary information

Supplementary information.
