# Author Correction: Effects of rainfall manipulation and nitrogen addition on plant biomass allocation in a semiarid sandy grassland

**DOI:** 10.1038/s41598-020-70621-x

**Published:** 2020-09-08

**Authors:** Jing Zhang, Xiaoan Zuo, Xueyong Zhao, Jianxia Ma, Eduardo Medina-Roldán

**Affiliations:** 1 Northwest Institute of Eco-Environment and Resources, Chinese Academy of Sciences, Lanzhou, 730000 China; 2 Health and Environmental Science Department, Xi’an Jiaotong Liverpool University, Suzhou, 215123 China

Correction to: *Scientific Reports* 10.1038/s41598-020-65922-0, published online 03 June 2020

This Article contains an error in Reference 41 which was incorrectly given as:

“Victoria, F. B., Jairo, A. P., Chen, Y. L. & Kadambot, H. M. Early season drought largely re-duces grain yield in wheat cultivars with smaller root systems. *Plants* **8**, 305 (2019).”

The correct Reference is listed below.

Figueroa-Bustos, V., Palta, J.A., Chen, Y. L. & Siddique, K.H.M. Early season drought largely reduces grain yield in wheat cultivars with smaller root systems. *Plants* **8**, 305 (2019).

