# Supplementary material for: Effects of rainfall manipulation and nitrogen addition on plant biomass allocation in a semiarid sandy grassland
Source: Sci Rep. 2020 Jun 3;10:9026. doi: 10.1038/s41598-020-65922-0 (PMC7270118; doi:10.1038/s41598-020-65922-0)
Supplement: Supplementary file 1 — Supplementary information. [file 41598_2020_65922_MOESM1_ESM.docx]

Effects of rainfall manipulation and nitrogen addition on plant biomass allocation in a semiarid sandy grassland

Jing Zhang^1^, Xiaoan Zuo^1*^, XueYong ZHAO^1^, Jianxia Ma^1^, Eduardo Medina-Roldán^2^

^1^ Northwest Institute of Eco-Environment and Resources, Chinese Academy of Sciences, Lanzhou, 730000, China

^2^ Health and Environmental Science Department, Xi'an Jiaotong Liverpool University, Suzhou, 215123, China

*Corresponding authors: E-mail: [zuoxa@lzb.ac.cn](mailto:zuoxa@lzb.ac.cn)

**Appendix**

Table S1 Vegetation characteristics under different treatments in 2017 and 2018

|  |  | Cover | | Species richness | | Plant total density | |
| --- | --- | --- | --- | --- | --- | --- | --- |
| Rainfall |  | 2017 | 2018 | 2017 | 2018 | 2017 | 2018 |
|  | -60% | 66.25±5.17 a | 59.08±1.96 b | 4.50±0.38 a | 6.42±0.42 a | 123.50±23.68 b | 85.67±16.66 b |
|  | CK | 84.58±2.08 a | 116.83±5.89 a | 4.50±0.26 a | 8.00±1.46 a | 162.12±23.18 b | 103.67±16.12 b |
|  | -60% | 84.38±2.92 a | 100.50±3.56 a | 5.00±0.53 a | 6.58±0.60 a | 212.00±53.37 a | 146.00±20.87 a |
| Nitrogen | CK | 77.33±2.07 a | 89.9±4.35 a | 4.96±0.27 a | 7.167±0.30 a | 204.67±27.15 a | 90.13±8.19 a |
|  | +N | 77.00±3.70 a | 89.67±4.28 a | 5.00±0.27 a | 6.63±0.31 a | 138.08±18.73 a | 121.6±12.13 a |

Table S2 The classification of life forms of different species in this study

| Life forms | Plant species |
| --- | --- |
| Annuals | *Echinops gmelini turcz.* |
|  | *Digitaria cilliaris(Retz.) Koel.Descr. Gram.* |
|  | *Chenopodium glaucum L.* |
|  | *Tribulus terretris L.* |
|  | *Tragus mongolorum Ohwi* |
|  | *Setaria viridis (L.) Beauv.* |
|  | *Eragrostis pilosa（L.）Beauv.* |
|  | *Artemisia scoparia Waldst. et Kit.* |
|  | *Erodium stephanianum Willd.* |
|  | *Salsola collina Pall.* |
|  | *Bassia dasyphylla (Fisch. et. Mey.) O. Kuntzae,Revis. Gen.* |
|  | *Panicum ruderale (Kitag.) Chang* |
| Perennials | *Pennisetum centrasiaticum Tzvel.* |
|  | *Cleistogenes squarrosa(Trin.) Keng* |
|  | *Cynanchum theisiodes(Freyn) K. Schum.* |
|  | *Lespedeza davurica(Laxm.)Schindl.* |
|  | *Phragmites communis Trrin. Fund.* |
|  | *Melissitus ruthenicus (L.) C.W.Chang* |
|  | *Allium mongolicum Rgl.* |
|  | *Convolvulus arvensis L.* |
